# Intervention by clinical pharmacists can improve blood glucose fluctuation in patients with diabetes and acute myocardial infarction: A propensity score‐matched analysis

**DOI:** 10.1002/prp2.725

**Published:** 2021-02-28

**Authors:** Fang‐Hong Shi, Long Shen, Jiang Yue, Jing Ma, Zhi‐Chun Gu, Hao Li, Hou‐Wen Lin

**Affiliations:** ^1^ Department of Pharmacy Renji Hospital, School of Medicine, Shanghai Jiaotong University Shanghai China; ^2^ Department of Cardiology Renji Hospital, School of Medicine, Shanghai Jiaotong University Shanghai China; ^3^ Department of Endocrinology Renji Hospital, School of Medicine, Shanghai Jiaotong University Shanghai China; ^4^ Department of Pharmacy, Shanghai Children's Medical Center Shanghai Jiao Tong University School of Medicine Shanghai China

**Keywords:** blood glucose, clinical pharmacist's intervention, consultation, glucose fluctuation, propensity score matching

## Abstract

Acute phase hyperglycemia and exaggerated glucose fluctuation may be associated with poor outcomes in diabetic patients after acute myocardial infarction (AMI). This study aimed to determine whether intervention by clinical pharmacists can mitigate blood glucose and glucose fluctuations in these fragile patients. This retrospective study enrolled patients with diabetes and AMI, from 1 January 2019 to 30 June 2020 in our institution. Blood glucose and glucose fluctuations were calculated before and after the pharmacist's intervention and between patients who underwent intervention and those who did not. Propensity score matching (PSM) was used to reduce the impact of patient characteristics on the results. A total of 170 patients were included in our primary analysis, including 29 patients who received the pharmacist intervention and 141 patients who did not. After the pharmacist's intervention, blood glucose (fasting blood glucose‐FBG, from 11.9 to 9.8; postprandial blood glucose‐PBG, from 15.3 to 13.2; mean blood glucose‐BG, 14.5 to 12.3 mmol/L; *p* < .001), and glucose fluctuations (standard deviation of blood glucose‐SDBG, from 3.8 to 3.0, mmol/L, *p* = .005) were significantly improved. Before PSM, no clear effects were found in intervention versus nonintervention patients, in terms of blood glucose and glucose fluctuation indicators, except for FBG (9.3 vs. 8.0. mmol/L, *p* = .005). Further analysis indicated a high incidence of FBG <7.8 mmol/L in nonintervention versus intervention patients (51.5% vs. 27.6%, *p* = .003). After PSM, a significant reduction in blood glucose fluctuation (SDBG, 3.0 vs. 4.1, *p* = .031; PBGE, 2.1 vs. 4.1, *p* = .017; LAGE, 4.7 vs. 7.2, mmol/L, *p* = .004), and PBG (11.1 vs. 13.0, mmol/L, *p* = .048) was observed in the intervention group than in the nonintervention group. The clinical pharmacist intervention contributed to improved outcomes, specifically, in reducing blood glucose fluctuations and potential hypoglycemia risk.

**What is already known about this subject:**

- Acute phase hyperglycemia and exaggerated glucose fluctuations may be associated with poor outcomes in diabetic patients after acute myocardial infarction (AMI). Clinical pharmacists can improve blood glucose in diabetic patients mainly through hemoglobin A1c (HbA1c), fasting blood glucose (FBG), and cardiovascular disease risk factors. However, current intervention of clinical pharmacists lacked the evaluation of blood glucose fluctuations.

**What this study adds:**

- Clinical pharmacist's intervention can mitigate blood glucose and glucose fluctuations in diabetes and acute myocardial infarction (AMI) patients in the hospital. Based on the glucose management goals of these fragile patients, pharmacist's intervention also may reduce potential hypoglycemia risk.

## 1 INTRODUCTION

Diabetes mellitus (DM) is an important and frequent comorbidity in patients with acute myocardial infarction (AMI). DM is being present in nearly 30% of AMI cases.^1^ AMI patients with DM have a significantly higher mortality than those without diabetes.^2^ Many studies have shown a relationship between high blood glucose levels on admission and an increased risk of mortality and poor outcomes after AMI. Studies further verified the association between glucose fluctuation during the phases of AMI and the extent of myocardial salvage.^3^ Rapid blood glucose fluctuation levels increase oxidative stress and are even more detrimental than sustained hyperglycemia. As a result, it is suggested that interventional trials in type 2 DM (T2DM) should not only focus on the blood glucose but also on acute glucose swings.^4^ Glucose fluctuations are also closely related to electrocardiographic surrogate markers of impaired myocardial salvage in AMI after reperfusion therapy,^5^ and have been shown to be correlate with endothelial dysfunction and atherosclerotic development.^4^ Accordingly, glucose fluctuation has been found to be a negative prognostic factor for patients with AMI,^6^ and may be a potential detrimental factor for salvaging ischemic damage.^3^ The reduction of glucose fluctuation may offer a potentially therapeutic strategy for decreasing myocardial reperfusion injury in AMI patients.^5^ Many components, including beta‐cell function, diet, exercise, and drugs, contribute to glucose fluctuations.^7^ Specifically, improper use of hypoglycemic drugs, poor adherence to medication by the patients, irregular food and exercise patterns, and nonstandard insulin injection will certainly cause glucose fluctuation.^7^, ^8^ Currently, clinical pharmacists play a vital role in blood glucose management within the diabetic care teams. Clinical pharmacists provide several clinical pharmacy services to promote the rational use of medications in diabetic patients, inducing a thorough evaluation of the patient status, providing suggestions on drug treatment to physicians, delivering relevant education to patients.^9^, ^10^ Compared to the usual care regime, pharmacist interventions have resulted in favorable improvements in the blood glucose indices including hemoglobin A1c (HbA1c), and fasting blood glucose (FBG), as well as other cardiovascular risk factors involving blood pressure, body mass index (BMI), total cholesterol, low‐density lipoprotein, high‐density lipoprotein, and triglycerides.^11^ However, evidence supporting the effects of clinical pharmacist interventions on the blood glucose fluctuations are limited. Therefore, this study aimed to evaluate the effects of a consultation model coordinated by clinical pharmacists and clinicians, on the blood glucose levels and glucose fluctuations in diabetic patients with AMI.

## 2 MATERIALS AND METHODS

### 2.1 Study population

AMI patients with DM, including ST‐segment elevation myocardial infarction (STEMI) and non‐ST‐segment elevation myocardial infarction (NSTEMI), admitted to our institution, from January 2019 to June 2020 were included. The inclusion criteria were as follows: (1) diagnosis of diabetes; (2) diagnosis of STEMI or NSTEMI; (3) length of hospital stay for more than 3 days; (4) available glucose monitoring data; (5) treatment with oral or intravenous hypoglycemic agents; and (6) aged 18 years or older. Patients were excluded if they had undergone lifestyle interventions or if glucose monitoring data were unavailable. This study included two parts. In the first part, clinical pharmacists carried out interventions for the patients with uncontrolled blood glucose and evaluated changes in the blood glucose and fluctuations after interventions. In the second part, comparison between patients who underwent pharmacist intervention and those who did not was performed using the propensity score matching (PSM) analysis. The study protocol was approved by the ethics committees of Renji Hospital, School of Medicine, Shanghai Jiaotong University (KY2019‐076) and informed consent was obtained from each patient or their family members.

### 2.2 The clinical pharmacist intervention model

**FIGURE 1:**
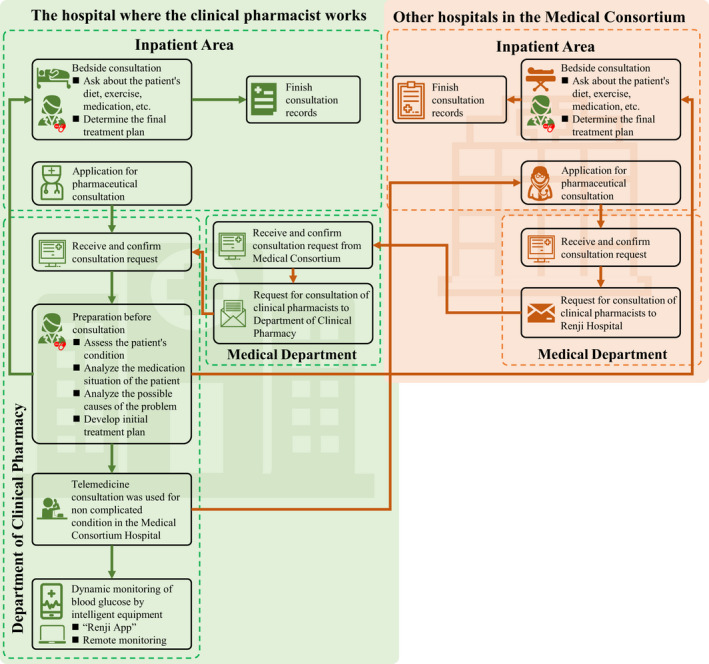
The flow diagram of the procedure of clinical pharmacist's consultation

Pharmacists working in the endocrinology department are skilled in glucose management of diabetic patients and have more than 5 years’ work experience. The pharmacist intervention was based on a model of consultations with the pharmacist, including both face to face and telemedicine consultations (Figure 1). The protocol for pharmacist consultations in the hospital where the clinical pharmacists worked was as follows: First, the clinicians applied for a consultation on the electronic system, then a receipt to confirm the pharmacist consultation request. Second, before each pharmacist consultation, the clinical pharmacists prepared for the consultation by assessing the patient's condition, analyzing the medications prescribed and any possible drug‐related problems, and developing an initial treatment plan. For complex conditions, the clinical pharmacists conducted face to face interviews with the patients to enquire about their diet, exercise regimen, medications, etc., and determine the final treatment plan. For noncomplicated conditions, the pharmacist consultation was conducted via telemedicine using the “Renji App” (mobile electronic case system). This system was also used by the pharmacists for dynamic monitoring of the blood glucose levels. The procedures of the pharmacist consultation in other hospitals where clinical pharmacists did not work appeared to be similar to the above process. These processes varied mainly in terms of whether there was a need for the medical department of the hospital to request for a clinical pharmacist consultation to Renji Hospital, and then for the medical department of the Renji Hospital to acknowledge the consultation request and issue a confirmation. Finally, following confirmation, the medical department then sent the request for a consultation by the clinical pharmacist of the department of clinical pharmacy.

### 2.3 Specific pharmacist interventions for controlling glucose fluctuations

The specific pharmacist interventions included consultation‐based health education and drug optimization. First, for each patient, the pharmacist conducted a custom‐designed health education list that involved four individualized dietary guidelines (food type, dining order, dining way, total quantity) and patient‐centered exercise guidelines (time, frequency, intensity, and types of sports). Second, the pharmacist optimized the patient's medication regimen based on the patient's baseline characteristics (weight, BMI, renal and liver functions, pancreas islet function, diabetic history, etc.) and the drug properties (influences on body weight, islet function, cardioprotective effect, hypoglycemia risks, adverse drug events, etc.), which may reduce the incidence of drug‐related problems (contraindicated drugs, hypoglycemia risk, infection risk, ketoacidosis risk, etc.). Finally, the pharmacist adjusted the patient's medication regimen according to their individual blood glucose targets and blood glucose dynamic changes during the hospital stay.

### 2.4 Data collection

Patient characteristics (demographics, diagnosis, and diabetes‐related indices) were recorded by reviewing the medical charts and hospital information system. The blood glucose levels were detected using Contour TS blood glucose meter (Bayer HealthCare) and Contour TS blood glucose test strips. Finger prick blood glucose data were used to evaluate the blood glucose fluctuations. The blood glucose data including fasting blood glucose (FBG, 6:00 am), post‐breakfast blood glucose (PBG, 9:00 am), post‐lunch blood glucose (PBG, 13:00 pm), and post‐dinner blood glucose (PBG, 19:00 pm) were obtained from the blood glucose test records in the electronic system. In the first part, glucose data were collected 4 days before and after the pharmacist consultation. In the second part, as part of the PSM analysis, the FBG data were collected 3 days before discharge and the PBG data were collected 24 h before discharge.

### 2.5 Outcomes measures

The outcomes included indicators for blood glucose and blood fluctuations. The blood glucose indicators were mainly composed of mean FBG, mean PBG, and mean BG. The blood fluctuation indicators included standard deviation of the blood glucose (SDBG), postprandial glucose excursion (PBGE), and largest amplitude of glycemic excursions (LAGE), according to the Chinese endocrinologist consensus on the management of glycemic variability of diabetes mellitus.^7^ The SDBG was calculated by changes in the blood glucose throughout one day, which assessed the extent to which the population deviated from the mean glucose level, but did not distinguish between maximum and minimum fluctuations. The PBGE was calculated as the mean value of the absolute difference between the postprandial blood glucose after three meals and pre‐meal blood glucose. The LAGE was calculated as the difference between the intraday maximum and minimum glycemic values. All three parameters were used to estimate the blood glucose fluctuations by means of self‐monitoring of the blood glucose (SMBG).

### 2.6 Data analyses

Continuous variables were described using mean with standard error (SE) and compared with the unpaired Student's *t*‐tests or Wilcoxon signed‐rank tests between the pharmacist intervention and nonintervention groups. Continuous variables before and after intervention were compared using paired Student's *t*‐tests. Categorical variables were expressed as counts and percentages and compared using the chi‐square test or Fisher's exact test, as appropriate. PSM was used to correct the differences in patient characteristics between the pharmacist intervention and nonintervention groups.^12^ Briefly, multiple logistic regression analysis was used to estimate the propensity score, which included the corrected variables (FBG, PBG, and length of hospital stay, etc.). The matching tolerance achieved by matching was assessed by calculating absolute standardized differences in covariates between the two groups, with 0.03 for measured covariates proposing appropriate balance. All statistical analyses were performed using SPSS 22.0 software (SPSS Inc.) and *p < *.05 was considered significant.

## 3 RESULTS

### 3.1 Patient characteristics

**FIGURE 2:**
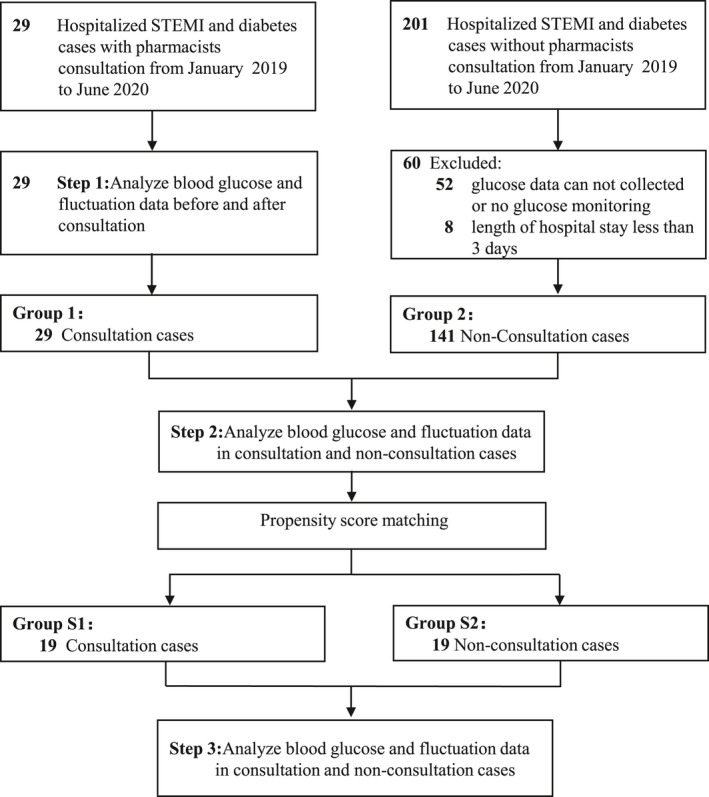
The flow diagram of the selection process to determine eligible individuals. STEMI: ST‐segment elevation myocardial infarction; NSTEMI: non‐ST‐segment elevation myocardial infarction

Figure 2 presents the flow diagram of this study. Totally, 29 patients received interventions based on the pharmacist consultations. In addition, a total of 201 hospitalized AMI and diabetes cases without intervention were reviewed from the electronic health records. Finally, 141 patients who did not undergo intervention were eligible for inclusion into this study. Of total 170 patients of both groups, the mean age of patients was 66.3 years. The percentage of male was 72.4%. The mean durations of diabetes were 9.3 years and mean HbA1c% was 8.1%. The mean blood glucose within 24 h of admission was elevated, with a mean FBG of 9.8 mmol/L and mean PBG of 13.5 mmol/L (Table 1).

**TABLE 1:** Characteristics of STEMI and diabetic patients in consultation or nonconsultation before and after PSM

Characteristics	Before PSM		After PSM	
	No consultation (*n* = 141)	Consultation (*n* = 29)	No consultation (*n* = 19)	Consultation (*n* = 19)
Age (year)	66.2 ± 0.9	67.0 ± 2.3	67.6 ± 2.9	67.0 ± 2.7
Male (%)	105 (74.5)	18 (62.1)	14 (73.7)	13 (68.4)
Female (%)	36 (25.5)	11 (37.9)	5 (26.3)	6 (31.6)
Body weight (kg)	69.7 ± 1.1	65.5 ± 2.6	71.4 ± 2.6	66.3 ± 3.2
BMI (kg/m^2^)	25.3 ± 0.3	24.3 ± 0.8	25.7 ± 0.6	24.3 ± 0.9
Diabetic data				
Diabetes duration (year)	9.4 ± 0.9	9.0 ± 1.6	8.4 ± 3.9	8.8 ± 2.0
HbA1c (%)	7.9 ± 0.1	9.0 ± 0.3**	8.9 ± 0.4	9.1 ± 0.4
FBG (mmol/L)	9.3 ± 0.3	12.3 ± 0.8**	11.0 ± 1.0	10.9 ± 0.7
PBG (mmol/L)	12.9 ± 0.3	16.5 ± 0.7**	14.7 ± 1.0	15.8 ± 0.7
Length of hospital stay (day)	7.7 ± 0.4	15.3 ± 2.7**	10.0 ± 1.8	10.0 ± 1.1

Blood glucose data collected from blood glucose monitoring from fasting blood glucose (FBG, 24 h within admission), post‐breakfast blood glucose (PBG, 9:00 am), post‐lunch blood glucose (PBG, 13:00 pm), and post‐dinner blood glucose (PBG, 19:00 pm). PBG were collected by average after three meals. BMI, body mass index, HbA1c%, glycosylated hemoglobin. Data were described as mean ± SE.

* *p* < .05.

** *p* < .01.

Baseline patient characteristics before and after PSM are presented in Table 1. Before PSM, the features of age, sex, body weight, body mass index (BMI), and durations of diabetes were comparable. However, in terms of the blood glucose indicators, compared with the nonintervention patients, the pharmacist intervention patients showed higher levels of HbA1c%, FBG, PBG, and longer length of hospital stay. After PSM, the above indicators were well balanced, resulting in 19 patients in each group.

### 3.2 Glucose levels and glucose fluctuations before and after the pharmacist intervention

**FIGURE 3:**
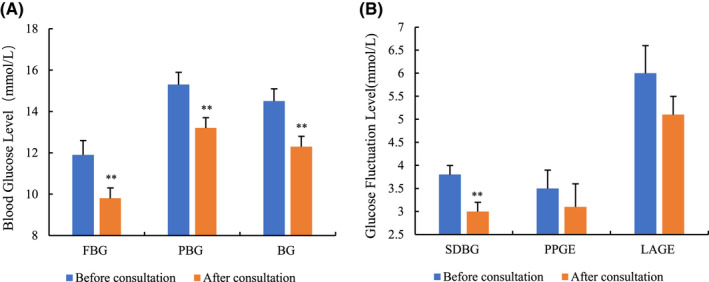
Blood glucose and fluctuation data before and after pharmacist consultation. Blood glucose (A) before and after pharmacist consultation, which contains FBG, PBG, and BG. Glucose fluctuation (B) before and after pharmacist consultation, which contain SDBG, PBGE, and LAGE. Blood glucose data collected from blood glucose monitoring from fasting blood glucose (FBG, 6:00 am), post‐breakfast blood glucose (PBG,9:00 am), post‐lunch blood glucose (PBG,13:00 pm), and post‐dinner blood glucose (PBG,19:00 pm). Continuous data were collected 4 days before and 4 days after pharmacist consultation. BG, blood glucose; SDBG, standard deviation of blood glucose; PBGE, postprandial glucose excursion; LAGE, largest amplitude of glycemic excursions. Data were described as mean ± SE, *p* < .05 was considered as a significant difference. ^**^ *p* < .01 and ^*^ *p* < .05

After the pharmacist intervention, the glucose indicators showed a significant improvement (mean FBG decreased from 11.9 mmol/L to 9.8 mmol/L; mean PBG from 15.3 mmol/L to 13.2 mmol/L; mean BG from 14.5 mmol/L to 12.3 mmol/L; *p* < .001 for each index) (Figure 3A). In terms of glucose fluctuation indices, the pharmacist intervention group showed significantly lower SDBG (3.8 mmol/L vs. 3.0 mmol/L, *p* = .005). Although there was no statistical difference, indicators, including PBGE and LAGE, illustrated a downward trend after the pharmacist intervention (PBGE, 3.53 mmol/L vs. 3.1 mmol/L, *p* = .461; LAGE, 6.0 mmol/L vs. 5.1 mmol/L, *p* = .201) (Figure 3B).

### 3.3 Glucose levels and glucose fluctuations in the intervention and nonintervention patients

**FIGURE 4:**
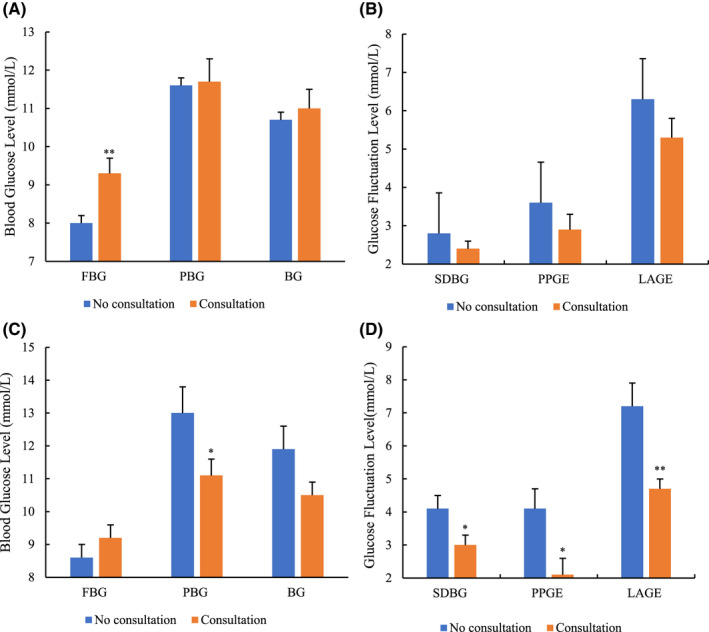
Blood glucose and fluctuation data before and after propensity score matching (PSM). Blood glucose (A) and fluctuation (B) data before PSM and Blood glucose (C), and fluctuation(D) data after PSM. Blood glucose mainly contains FBG, PBG, and BG and blood fluctuation mainly contains SDBG, PBGE, and LAGE. Blood glucose data collected from blood glucose monitoring from fasting blood glucose (FBG, 6:00 am), post‐breakfast blood glucose (PBG, 9:00 am), post‐lunch blood glucose (PBG,13:00 pm), and post‐dinner blood glucose (PBG,19:00 pm). FBG data were collected 3 days before discharge and PBG data were collected 24 h before discharge and fluctuation data were calculated mainly based on 24 h before discharge. BG, blood glucose; LAGE, largest amplitude of glycemic excursions; PBGE, postprandial glucose excursion; SDBG, standard deviation of blood glucose. Data were described as mean ± SE, *p* < .05 was considered as a significant difference. ^**^ *p* < .01 and ^*^ *p* < .05

Before PSM, as for the glucose indicators, there were no significant differences between the intervention and nonintervention patients in terms of mean PBG (11.7 mmol/L vs. 11.6 mmol/L, *p* = .841) and mean BG (11.0 mmol/L vs. 10.7 mmol/L, *p* = .542). Unexpectedly, we found a seemingly opposite result. The mean FBG was higher in the intervention group than in the nonintervention group (9.3 mmol/L vs. 8.0 mmol/L, *p* = .005) (Figure 4A). According to the patient's condition and the reasons for admission, the glycemic goals of AMI patients should be set to less stringent levels, for example, the level of AMI patients, FBG 7.8–10 mmol/L and PBG 7.8–13.9 mmol/L, based on Chinese endocrinologist consensus on blood glucose management for Chinese inpatients.^13^ As such, FBG <7.8 mmol/L may be considered as a potential risk for hypoglycemia in patients with AMI, even though hypoglycemia did not occur in these patients.

After further analysis, we found a higher incidence of FBG<7.8 mmol/L in the nonintervention patients than in the intervention patients (218/423, 51.5% vs. 24/87, 27.6%, *p* = .003). There were no statistically significant differences between the two groups among those who actually reached the target (130/423, 30.7% vs. 30/87, 34.5%, *p* = .648) (Table 2). In addition, similar results between the intervention and nonintervention patients were observed in the glucose fluctuation indices, such as SDBG (2.4 mmol/L vs. 2.8 mmol/L, *p* = .176), PBGE (2.9 mmol/L vs. 3.6 mmol/L, *p* = .176), and LAGE (5.3 mmol/L vs. 6.3 mmol/L, *p* = .176) (Figure 4B).

**TABLE 2:** Fasting blood glucose stratify according to blood glucose standards before propensity score matching

Blood glucose indexes	No intervention (*n* = 141)	Intervention (*n* = 29)	*p* value
FBG<7.8 mmol/L (day)	1.5 ± 0.1	0.8 ± 0.2	.003
FBG<7.8 mmol/L (%)	51.5 (218/423)	27.6 (24/87)	
FBG 7.8–10 mmol/L (day)	0.9 ± 0.1	1.0 ± 0.2	.648
FBG 7.8–10 mmol/L (%)	30.7 (130/423)	34.5 (30/87)	

Blood glucose data collected from blood glucose monitoring from fasting blood glucose (FBG, 6:00 am), FBG data were collected 3 days before discharge. Frequency of FBG<7.8 mmol/L and FBG 7.8–10 mmol/L in 3 days before discharge. Data were described as mean ± SE, *p* < .05 was considered as a significant difference.

After PSM, the PBG was significantly lower in the intervention patients than in the nonintervention patients (11.1 mmol/L vs. 13.0 mmol/L, *p* = .048). However, when considering other glucose indices, such as mean FBG (9.2 mmol/L vs. 8.6 mmol/L, *p* = .267) and BG (10.5 mmol/L vs. 11.9 mmol/L, *p* = .105), no clear effects were found between the two groups (Figure 4C). Of note, a significant effect of glucose fluctuation reduction was observed among the pharmacist intervention patients. Specifically, the pharmacist intervention patients had decreased SDBG (3.0 mmol/L vs. 4.1 mmol/L, *p* = .031), PBGE (2.1 mmol/L vs. 4.1 mmol/L, *p* = .017), and LAGE (4.7 mmol/L vs. 7.2 mmol/L, *p* = .004) than the nonintervention patients (Figure 4D).

## 4 DISCUSSION

Our study focused on a new pharmacist–clinician collaboration model based on the addition of pharmacist consultations to the AMI patients with DM, with the goal of optimizing comprehensive care for these patients. In this study, the pharmacist intervention was associated with improvements in the blood glucose levels (mean FBG, mean PBG, and mean BG) as well as in glucose fluctuations (SDBG, PBGE, and LAGE). Furthermore, the clinical pharmacists were able to reduce the potential risk of hypoglycemia and make the blood glucose control more achievable for these frail patients.

Clinical pharmacists practicing in medical institutions are easily accessible and can play a vital role in providing timely advice for diabetic patients and therapeutic advice for the interdisciplinary care team.^14^ The most effective components of the pharmacist intervention were patient‐centered services and multidisciplinary care.^15^ A German study^16^ reported that pharmacists who provided independent case management were more effective when collaborating with clinicians within a multidisciplinary team. In this study, we explored a new pharmacist–clinician collaboration model based on pharmacist consultations, and found that the blood glucose levels and glucose fluctuations of diabetic patients were significantly improved through the pharmacist interventions model.

The rising mortality risk in patients with diabetes during and after AMI is of critical concern. There is an urgent need for better treatment choices in these patients in addition to intensive medications.^1^ Stringent control of risk factors, such as blood glucose, may be a good long‐term surveillance strategy in diabetic patients with AMI.^17^ The mean BG is an important predictive factor for in‐patient mortality, and has been indicated to be independently related to mortality in critically ill patients.^18^ A previous umbrella meta‐analysis reported that the pharmacist interventions mainly resulted in favorable improvements in HbA1c, FBG, and other cardiovascular risk factors.^11^ In our study, we not only assessed FBG, PBG, and mean BG before and after the pharmacist consultations, but also evaluated these factors between the pharmacist intervention and nonintervention patients. We found that the blood glucose indices (FBG, PBG, and mean BG) were significantly reduced after the pharmacist intervention. In order to further verify our findings, we used the PSM method to control for multiple patient factors. The PSM result showed that the blood glucose indices improved with the pharmacist interventions.

Glycemic disorders, including diabetes, impaired glucose tolerance and stress hyperglycemia are commonly seen in patients with AMI.^19^ Glycemic disorder is considered a major predictor of poor clinical outcomes in AMI patients, which is not only limited to constant hyperglycemia, but also involves glucose fluctuations.^20^, ^21^ Most previous studies mainly concentrated on HbA1c and other cardiovascular risk factors after AMI.^11^, ^22^ There is a lack of assessment of the blood glucose fluctuations for patients with AMI. Current evidence support that glucose fluctuation parameters are closely related to electrocardiographic surrogate markers of injured myocardial salvage in AMI after reperfusion therapy.^21^ Some drugs, such as miglitol, can mitigate glucose fluctuations with subclinical hypoglycemia through alterations of heart rate variability and the sympathetic nervous system in T2DM patients with recent acute coronary syndrome.^23^ Our study indicated that the pharmacist intervention comprehensively improved three glucose fluctuation factors among diabetic patients with AMI. The beneficial effect of the pharmacist intervention on glucose fluctuation was encouraging because we not only evaluated glucose fluctuation improvement after the pharmacist intervention, but also applied PSM to assess improvement of glucose fluctuations in the pharmacist intervention patients compared with nonintervention patients. Other studies^8^, ^24^ have also suggested that many factors, such as diet, exercise, islet function, and medications influence glucose fluctuation, and that pharmacists may play a key role in the above factors. In brief, after the pharmacist intervention, the pharmacists can update the physicians regarding the blood glucose status in these patients, educate patients to pay specific attention to the relevant areas, such as their diet, exercise, and blood glucose monitoring and assist with the selection of drugs that are more appropriate for the individual patients.

Meanwhile, concerning the harmful effect of hyperglycemia in patients with AMI, the blood glucose target goals in these patients is important. Different blood glucose control targets should be set for hospital patients with different health conditions, based on the Chinese endocrinologist consensus on blood glucose management for Chinese inpatients.^13^ According to a patient's condition and the reasons for admission, the glycemic goals of AMI patients should be set to a less stringent level, indicating FBG 7.8–10 mmol/L and PBG 7.8–13.9 mmol/L. STEMI patients with silent hypoglycemia have an obviously higher frequency of ventricular extrasystoles and nonsustained ventricular tachycardias.^25^ Therefore, reducing unnecessary hypoglycemia is crucial for patients with AMI. Our experiences of proper glucose management of these patients were published in a previous study.^26^ The proportion of patients who experienced potential hypoglycemia risk (FBG <7.8 mmol/L) was reduced from 50% to 26.7% after the pharmacist intervention in this study. Although there was no report of hypoglycemia events in the nonintervention patients, the probable potential risk still needs further attention.

In this study, we applied the new model of glucose management coordinated by clinical pharmacists and clinicians, which we reported at length in our previous study.^26^ In China, clinical pharmacists play a vital role in clinical medication and patient management. An increasing number of specialist clinical pharmacists is involved in clinical treatment, after 1 year of professional training at National Clinical Pharmacist Training Bases. Regarding blood glucose management, more than 5 years of endocrine work experience is required. Specialist clinical pharmacists work in nonendocrine clinical departments and participate in the selection and adjustment of medication treatment. Besides clinical pharmacist consultations, another core management strategy involves pharmacist‐mediated medication education programs. Clinical pharmacists can greatly assist in supporting physicians by making up for the doctor's lack of time,^27^ a perceived lack of receptivity,^28^ and possibly, poor knowledge of some specific drugs.^29^

### 4.1 Strengths and limitations

The strengths of this study relate to the pharmacist intervention in diabetic patients with AMI. Professional endocrine clinical pharmacists not only designed the medical schedule, but also monitored the implementation of the plan, followed up with patients, and continuously optimized the scheme. The PSM analysis was also strength. Given that the baseline characteristics (including HbA1c%, FBG, PBG, and longer length of hospital stay) were not comparable between the pharmacist intervention and nonintervention groups, the PSM method was used to reduce the confounding factors, resulting in 19 patients in each group. Several limitations in this preliminary study need to be considered. First, this was a single‐center study. Second, the sample size was insufficient. Therefore, further studies with larger sample sizes and multiple centers are needed to strengthen the conclusion that the pharmacist intervention model improves blood glucose management. Furthermore, this model was applied mainly within the department of cardiology and cardiac care unit in our institution. The extrapolation of this model can be further standardized by establishing an application (app) or WeChat program. Third, although we found that the pharmacist intervention was beneficial in controlling blood glucose management and mitigating glucose fluctuation, we did not include clinical events in this study. Finally, we calculated the indices of the blood glucose fluctuation using finger‐prick blood glucose data. A previous study^30^ suggested that the traditional finger‐prick blood glucose level monitoring provided a relatively reasonable approximation of the mean blood glucose concentration in most patients. However, this may underestimate the prevalence of potential hyperglycemia and hypoglycemia. Therefore, in future studies, the use of continuous blood glucose monitoring is recommended and may further verify our conclusions.

## 5 CONCLUSION

In this study, we found that the pharmacist intervention improved the blood glucose levels (mean FBG, mean PBG, and mean BG) as well as glucose fluctuations (SDBG, PBGE, and LAGE). Furthermore, clinical pharmacists may reduce the potential risk for hypoglycemia and make the blood glucose control more achievable for diabetic patients with AMI. The treatment model coordinated by clinical pharmacists and the clinicians may be recommended for blood glucose control, especially for patients with cardiovascular complications. This collaborative treatment model, involving clinical pharmacists and clinicians, should be the trend for future developments. Experiences based on this preliminary study were limited to a specific group of patients, but this study may serve as an example of a promising approach for blood glucose management.

## CONFLICT OF INTEREST

The authors have declared no conflicts of interest for this article.

## ETHICS APPROVAL STATEMENT

This study protocol was approved by ethics committees of Renji Hospital, School of Medicine, Shanghai Jiaotong University (KY2019‐076).

## PATIENT CONSENT STATEMENT

Informed consent was obtained from each patient or their family members.

## PRINCIPAL INVESTIGATOR STATEMENT

The authors confirm that the principal investigator for this paper is Fang‐Hong Shi and Zhi‐Chun Gu and that both of them had direct clinical responsibility for patients.

## Data Availability

The raw data supporting the conclusion of this article will be made available by the authors to related qualified researcher.
